# Organization and evolution of two SIDER retroposon subfamilies and their impact on the *Leishmania *genome

**DOI:** 10.1186/1471-2164-10-240

**Published:** 2009-05-22

**Authors:** Martin Smith, Frédéric Bringaud, Barbara Papadopoulou

**Affiliations:** 1 Research Centre in Infectious Diseases, CHUL Research Centre, RC-709, 2705 Laurier Blvd, Quebec (QC), G1V 4G2 Canada; 2 Department of Medical Biology, Faculty of Medicine, Laval University, Quebec, Canada; 3 Centre de Résonance Magnétique des Systèmes Biologiques, UMR-5536 CNRS, Université Victor Segalen Bordeaux 2, 146 rue Léo Saignat, 33076 Bordeaux, France

## Abstract

**Background:**

We have recently identified two large families of extinct transposable elements termed Short Interspersed DEgenerated Retroposons (SIDERs) in the parasitic protozoan *Leishmania major*. The characterization of SIDER elements was limited to the SIDER2 subfamily, although members of both subfamilies have been shown to play a role in the regulation of gene expression at the post-transcriptional level. Apparent functional domestication of SIDERs prompted further investigation of their characterization, dissemination and evolution throughout the *Leishmania *genus, with particular attention to the disregarded SIDER1 subfamily.

**Results:**

Using optimized statistical profiles of both SIDER1 and SIDER2 subgroups, we report the first automated and highly sensitive annotation of SIDERs in the genomes of *L. infantum, L. braziliensis *and *L. major*. SIDER annotations were combined to *in-silico *mRNA extremity predictions to generate a detailed distribution map of the repeat family, hence uncovering an enrichment of antisense-oriented SIDER repeats between the polyadenylation and *trans*-splicing sites of intergenic regions, in contrast to the exclusive sense orientation of SIDER elements within 3'UTRs. Our data indicate that SIDER elements are quite uniformly dispersed throughout all three genomes and that their distribution is generally syntenic. However, only 47.4% of orthologous genes harbor a SIDER element in all three species. There is evidence for species-specific enrichment of SIDERs and for their preferential association, especially for SIDER2s, with different metabolic functions. Investigation of the sequence attributes and evolutionary relationship of SIDERs to other trypanosomatid retroposons reveals that SIDER1 is a truncated version of extinct autonomous *ingi*-like retroposons (DIREs), which were functional in the ancestral *Leishmania *genome.

**Conclusion:**

A detailed characterization of the sequence traits for both SIDER subfamilies unveils major differences. The SIDER1 subfamily is more heterogeneous and shows an evolutionary link with vestigial DIRE retroposons as previously observed for the *ingi*/RIME and L1Tc/NARTc couples identified in the *T. brucei *and *T. cruzi *genomes, whereas no identified DIREs are related to SIDER2 sequences. Although SIDER1s and SIDER2s display equivalent genomic distribution globally, the varying degrees of sequence conservation, preferential genomic disposition, and differential association to orthologous genes allude to an intricate web of SIDER assimilation in these parasitic organisms.

## Background

*Leishmania *are parasitic protozoa transmitted by the bite of phlebotomine sandflies that are endemic to tropical and subtropical climates worldwide. More than 20 species of *Leishmania *cause a wide range of human diseases that range from self-healing cutaneous lesions (*L. major*/*L. tropica*/*L. mexicana*) to fatal visceral leishmaniasis (*L. donovani*/*L. infantum*/*L. chagasi*), mucosal leishmaniasis (mainly *Leishmania *(Viannia) *braziliensis*), and diffuse cutaneous leishmaniasis (mainly *L. amazonensis*/*L. guyanensis*/*L. aethiopica*) [1]. Leishmaniasis currently threatens 350 million people in 88 countries. It is estimated that 2 million new cases occur each year, with at least 12 million people presently infected worldwide [2,3]. Recent reports also indicate that leishmaniasis is now an emerging zoonosis in the United States [4-6]. Hope for discovering novel drug and vaccine targets stem from the recently fully sequenced genomes of several *Leishmania *species [7,8].

As opposed to higher eukaryotes, *Leishmania *and other kinetoplastids seem to have lost or never acquired the ability to regulate transcription initiation by RNA polymerase II [9,10]. Transcription has been postulated to initiate on each chromosome at divergent Strand-Switch regions (dSS); the 0.9- to 14-kb non-coding regions preceding opposite strand Directional Gene Clusters (DGCs) [10-12]. These locations display skewed sequence composition which may be functionally relevant to transcription initiation [13]. Typically, genes are grouped together into long, same strand polycistronic clusters where individual mRNAs are processed by *trans*-splicing and polyadenylation (reviewed in [14,15]). Although there is evidence for antisense transcription in *Leishmania *(reviewed in [16]), nuclear run-on studies have demonstrated that transcriptional orientation is controlled by termination (i.e., RNA polymerase II promptly aborting transcription in antisense orientation) [17]. It has also been shown that convergent strand-switch (cSS) regions may be involved in transcriptional termination [11]. In light of these observations, it comes as no surprise that regulation of gene expression occurs largely at the post-transcriptional level in these parasites. The discovery of many RNA-binding protein domains [9,18], regulated processing of cytoplasmic RNAs [19], and conserved regulatory elements in 3' untranslated regions (3'UTRs) [14,20-23] are hard evidence that corroborate this statement. Several studies have shown that *cis*-acting sequences within 3'UTRs regulate differential expression of the upstream gene mainly by modulating mRNA stability [14,21,24] or translational efficiency [23,25-28], although other mechanisms may exist [14,24].

To date, retroposons and long terminal repeat retrotransposons are the only Transposable Elements (TEs) that have been described in *Leishmania *and trypanosome genomes. These class I TEs compose up to 5% of trypanosomatid genomes [3,7,29]. *Leishmania major *and *L. infantum *are believed to lack potentially active TEs, such as *ingi *clade retroposons present in *Trypanosoma brucei *(*ingi*) and *T. cruzi *(L1Tc) [30]. However, the TE-derived highly Degenerated *ingi*/L1Tc-Related Elements (DIREs) have been reported in certain *Leishmania *species [31,32]. Recently, small degenerated retroposons (~0.55 kb) termed SIDERs (Short Interspersed DEgenerated Retroposons) have been identified in the genome of trypanosomes and *Leishmania *via iterative pairwise BLAST queries and manual annotation [21]. These extinct repeats are apparently related to the *ingi *clade of retroposons, do not display apparent site-specificity for genomic integration, and are preponderantly distributed in the intergenic regions of DGCs. SIDERs represent the most abundant TE family described in trypanosomatid genomes to date and can be divided into two subfamilies, namely SIDER1 and SIDER2, which present similar yet distinguishable sequence traits. A truncated portion of the antisense SIDER2 consensus was initially described as RS2 dispersed repeats in *L. major *mini-chromosomes by Ortiz and Segovia [33]. SIDER2 repeats have since been identified in *L. infantum *[34] via a methodology highly similar to the one employed by Bringaud *et al*. [21].

This work aims at further improving the characterization of the highly abundant family of extinct SIDER retroposons in a full-scale comparative genomics framework. We present the first global characterization of both SIDER1 and SIDER2 elements via optimized statistical profiles and sensitive automated annotation in three sequenced *Leishmania *species responsible for distinct pathologies. This enabled the comparison of both subgroups with other trypanosomatid retroposons, hence unveiling their evolutionary relation. Also, the distribution of SIDER elements within *Leishmania *genomes and among orthologous genes demonstrates that these extinct retroposons are not randomly distributed. Various hypotheses entailed by this investigation are set forth as we discuss possible functional and evolutionary implications of the broad assimilation of SIDER elements.

## Results

### Optimized alignment and automated annotation of SIDERs

**Figure 1:**
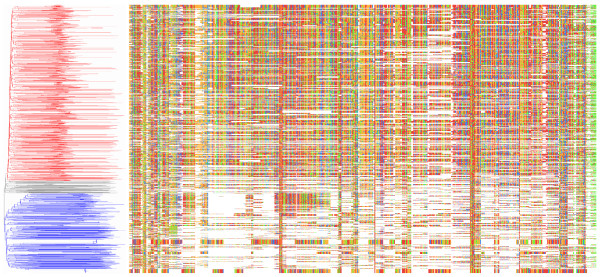
**Core alignment and associated phylogeny of 1416 *Lm*SIDER sequences**. All previously annotated *Lm*SIDER sequences encompassing 350–700 nt were extracted and submitted to HMM profile training using the simulated annealing and Baum-Welch expectation maximization algorithms implemented in *HMMER*-*1.8.5 *[65]. Columns containing more than 75% gap characters and failing to present a 10% consensus residue were removed to ease representation. The associated minimal evolution phylogeny, which was scaled to the alignment overview, was inferred with *MEGA4 *[66] based on p-distance and considering pairwise deletions. The red and blue clusters correspond to SIDER2 and SIDER1 sequences, respectively, whereas the grey cluster contains equal proportions of both SIDERs. Nucleotide composition is as follows: green = adenine; red = thymine; blue = cytosine; orange = guanine. The individual phylogeny and full alignment can be viewed in Additional file 1.

In order to fully characterize both SIDER subgroups in the genomes of *L. major, L. infantum*, and *L. braziliensis*, we sought an efficient and automated method capable of producing reliable alignments when confronted with sequences displaying variable rates of conservation. To date, the only detailed characterization of SIDER sequences is a rigorous manual alignment of 1013 *L. major *(*Lm*) SIDER2s set forth by Bringaud *et al*. [21], while the SIDER1 subgroup has yet to be properly portrayed. The scant sequence homology and size polymorphism of these degenerated retroposons make for irresolute multiple alignments, particularly with regards to the very heterogeneous SIDER1 subgroup. By applying metaheuristics for global parameter optimization and maximum likelihood estimators to a Hidden Markov Model (HMM) representation of SIDER sequences (see Methods), we generated a high quality alignment comparable to that published for *Lm*SIDER2 [21]. This approach allows for unbiased classification of SIDER subgroups when considering the underlying phylogenetic tree (Figure 1 and Additional File 1).

It is apparent that SIDER elements form two phylogenetically distinct groups when glancing at the global sequence composition of the two main clusters in Figure 1. To further appreciate subgroup-specific traits, both major phylogenetic subgroups of *Lm*SIDERs were separated and submitted to independent alignment optimization. Once filtered to remove highly homologous sequences (>90% sequence identity), the relative specificity of the initial profiles was tested by scanning all included *Lm*SIDER sequences with both HMM profiles using a global alignment algorithm (Additional file 2). Improperly labeled sequences (one SIDER1 and 29 SIDER2s) were swapped to their appropriate profiles that were finally resubmitted to the same optimization methodology. The resulting HMM profiles were used for pangenomic queries which include the initial scans for *L. infantum *and *L. braziliensis*. Results from the latter were submitted to one more round of optimization to generate the final refined profiles.

Since version 1.8.5 of *HMMER *software does not assign expectation values (E-values) to its predictions, we scanned a randomized synthetic genome of 50 Mb composed of similar nucleotide frequencies (e.g., 40% A+T, 60% C+G) so as to ascertain the specificity of each HMM profile. A bit-score threshold of 5 limited false positives to one or two at most for each profile. All false-positives scored less than 6 with an average of ~1.5 bits (data not shown). Applying this threshold to all final predictions is forthright given the reasonably smaller size of *Leishmania *genomes (~32 Mb) in comparison to the synthetic control.

### Sequence-based characterization of the SIDER1 subgroup

**Figure 2:**
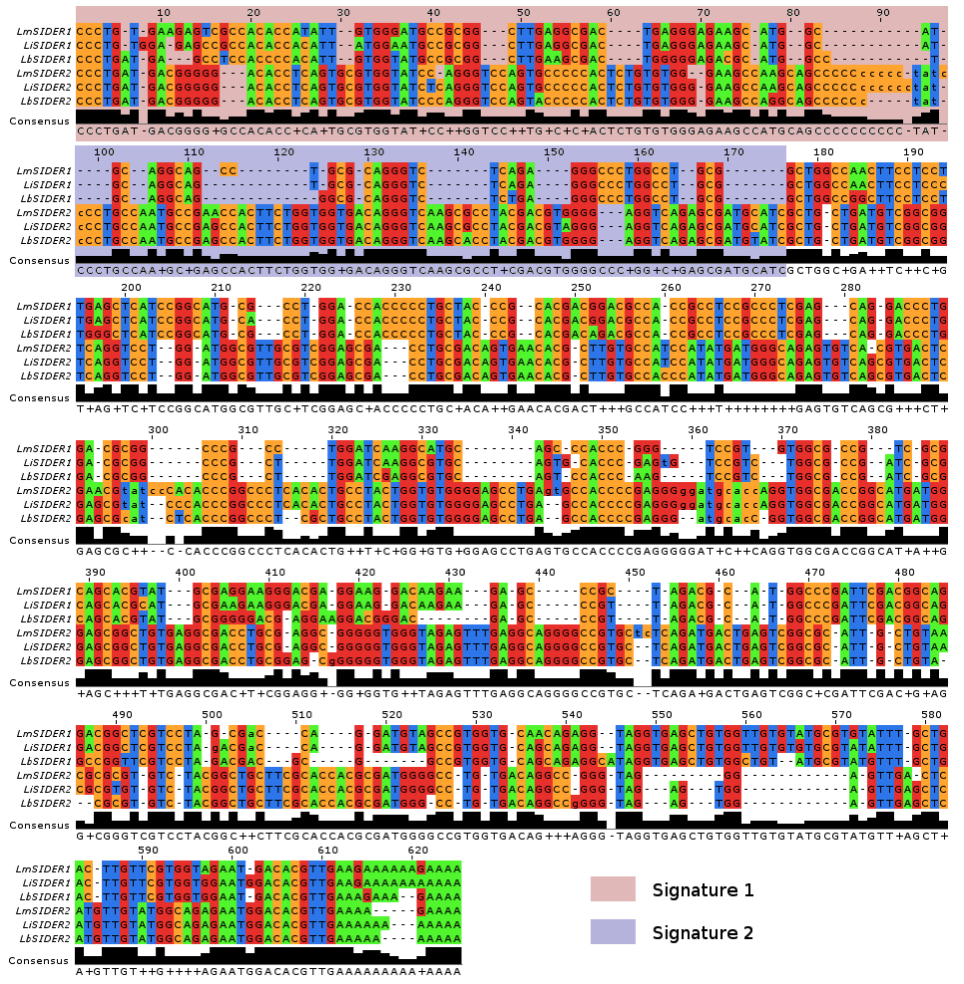
***Leishmania *spp. SIDER1 and SIDER2 consensus sequences**. Pairwise alignment of the most probable sequence for all refined SIDER profiles derived from independent HMM training for both phylogenetic clusters (see Methods section for details). The virtual positions of trypanosomatid retroposon signature sequences are highlighted in the pink and blue boxes. The black consensus bars highlight conserved positions. *JALVIEW *[8] was used to represent the alignment. Lm, *L. major*; Li, *L. infantum*; Lb, *L. braziliensis*.

Consensus sequences were extracted from the final refined profiles for both SIDER subgroups and compared among the three species (Figure 2). It is apparent that the SIDER1 cluster is riddled with deletions when compared to the SIDER2 cluster, though the consensus of both subgroups share 48% sequence similarity. Interestingly, SIDER1s appear to lack most of the second hallmark sequence of trypanosomatid retroposons (Figure 2, blue box) and present G/T-rich insertions at positions 545–570 when compared to SIDER2 consensus.

**Figure 3:**
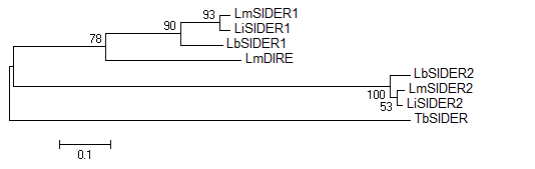
**Evolutionary relationship of trypanosomatid degenerated retroposons**. Minimal evolution phylogeny of the most probable sequence emitted from the HMM profile of degenerated retroposon classes (SIDER1 and 2 subfamilies, *Lm*DIRE and *Tb*SIDER). The phylogeny was based on maximum composite likelihood, assuming a heterogeneous substitution rate among lineages and completing deletions. Numbers represent bootstrap values, expressed as percentage, after 2000 replications and the *Tb*SIDER sequence was used to root the tree.

To further understand the evolutionary origins of SIDER elements, we compared their sequence composition to that of the *T. brucei *SIDERs (*Tb*SIDERs) and the *L. major *Degenerate *ingi*/L1Tc-Related Elements (*Lm*DIREs). Given their phylogenetic relationship to *Tb*SIDERs (Figure 3), *Leishmania *SIDERs have seemingly diverged a long time ago, conceivably after the emergence of the genus. Conversely, SIDER1 and SIDER2 subgroups apparently diverged prior to *Leishmania *speciation, as both retroposons form separate monophyletic groups. It is also evident that SIDER1s are related to *Lm*DIREs (Figure 3). This evolutionary relationship is confirmed by the comparative genomic distribution of *Lm*DIRE and *Lm*SIDER1 sequences. Indeed, 46 of the 51 annotated *Lm*DIREs overlap or are in close proximity to *Lm*SIDER1 sequences (Additional file 3. This clearly indicates that SIDER1 elements are related to the long and potentially coding DIREs, a situation comparable to the RIME/*ingi *and NARTc/L1Tc couples in *T. brucei *and *T. cruzi*, respectively. However, no *Lm*SIDER2 sequences are located in the vicinity of identified *Lm*DIREs. These observations suggest that the *Lm*SIDER1 subgroup corresponds to a truncated version of *Lm*DIREs that has spread throughout *Leishmania *genomes via the retrotransposition machinery encoded by active autonomous retroposons of the *ingi *clade (probably an ancestral active version of *Lm*DIRE). The expansion of *Lm*SIDER1 elements stopped when the active autonomous *ingi*-like retroposons disappeared from the ancestral *Leishmania *genome and became the present day *Lm*DIRE vestiges.

### Intra- and inter-species chromosomal distribution of SIDER subgroups

The species- and subgroup-specific optimized HMM profiles were used to annotate the final positions of SIDER elements in *Leishmania *genomes (Additional file 4). Both classes combined, we identified 1853 *Lm*SIDERs (Table 1), an amount which correlates with the initial estimate of 1858 identified with *BLASTN *and manual annotation [21]. In addition, we identified 1976 *L. infantum *SIDERs (*Li*SIDERs) and 1986 *L. braziliensis *SIDERs (*Lb*SIDERs). Our investigation suggests that the proportion of *Lm*SIDER1/*Lm*SIDER2 sequences is greater than previously proposed (785:1073 vs. 648:1205). *L. infantum *and *L. braziliensis *ostensibly harbor more SIDER1 sequences than *L. major *(740 and 721 vs. 648, respectively).

**Table 1:** Genomic distribution of SIDER elements among *Leishmania *species

**Species**	***L. major***		***L. infantum***		***L. braziliensis***	
			
	SIDER1 (%)	SIDER2 (%)	SIDER1 (%)	SIDER2 (%)	SIDER1 (%)	SIDER2 (%)
CDS						
Overlapping 5'ORF	0 (0.0)	7 (0.6)	4 (0.5)	7 (0.6)	1 (0.1)	5 (0.4)
Overlapping 3'ORF	0 (0.0)	5 (0.4)	3 (0.4)	4 (0.3)	3 (0.4)	1 (0.1)
Embedded in ORF	3 (0.5)	4 (0.3)	21 (2.8)	13 (1.1)	9 (1.2)	30 (2.3)
"Spacer" IR^a^						
Sense	28 (4.3)	43 (3.6)	62 (8.4)	38 (3.1)	44 (6.1)	28 (2.2)
Antisense	126 (19)	138 (11)	110 (15)	146 (12)	88 (12)	161 (13)
3'UTR^b^						
Sense	398 (61)	816 (68)	420 (57)	753 (61)	494 (69)	836 (66)
Antisense	54 (8.3)	64 (5.3)	27 (3.6)	62 (5.0)	27 (3.7)	62 (4.9)
Partial 3'UTR^ab, c^	25 (3.9)	65 (5.4)	58 (7.8)	136 (11)	20 (2.8)	66 (5.2)
Strand-switch^d^						
Convergent	6 (0.9)	27 (2.2)	13 (1.8)	37 (3.0)	15 (2.1)	30 (2.3)
Divergent	8 (1.2)	36 (3.0)	14 (1.9)	37 (3.0)	16 (2.2)	40 (3.2)
Subtelomeric	0 (0.0)	1 (0.1)	8 (1.0)	4 (0.3)	5 (0.7)	6 (0.5)

Total number	648	1205	740	1236	721	1265
	1853		1976		1986	

^a ^SIDER sequences between the polyadenylation site and the *trans*-splicing site.

^b ^As predicted with *PRED-A-TERM*.

^c ^Polyadenylation site predicted within SIDER element.

^d ^Interstitial, non-coding regions that characterize a changing orientation of directional gene clusters.

The results of the aforementioned SIDER search were combined to 3'UTR predictions generated with *PRED-A-TERM*, a *Leishmania*-specific mRNA extremity prediction algorithm [35], in order to ascertain the relative genomic dissemination of SIDER elements (Table 1). It has been shown that *Lm*SIDER2s are preponderantly positioned in the intergenic regions of directional gene clusters and that most of these elements (73%) reside within 3'UTRs [21]. However, such estimates are derived from mRNA extremity predictions based on an algorithm for the *Trypanosoma *genus [36]. As detailed in Table 1, all three sequenced *Leishmania *genomes withhold similar proportions of SIDERs predicted in 3'UTRs (73.7–77%). On average, 76% of SIDERs located in intergenic regions are enclosed in 3'UTRs, at least partially, when considering all possibilities (i.e., sense, antisense and partial). It is quite evident that SIDERs contained within 3'UTRs are enriched in the sense orientation (5'→3' in RNA) for all three species. This observation correlates with the total proportion of SIDERs in DGCs; sense SIDERs are typically 4-fold more abundant than antisense ones. Given the excess of SIDERs situated in the same orientation as directional gene clusters, one would assume that SIDER elements located in "spacer" intergenic regions (sequences between the polyadenylation site and the *trans*-splicing site) should display similar statistics. Quite the contrary, antisense SIDERs are on average over 3-fold more abundant than sense-oriented ones in these interstitial regions. Remarkably, antisense SIDERs that map to these regions do not display appreciably lower bit-scores, which would be expected for sequences that are not subjected to purifying selection.

In spite of this, not all SIDER elements are limited to intergenic regions. A significant number of SIDER elements map to strand-switch regions (i.e., the interstitial non-coding regions that characterize a changing orientation of DGCs). These chromosomal locations harbor 4–5% of all identified SIDERs, which are seemingly evenly distributed between divergent (dSS) and convergent (cSS) strand-switch regions. However, a slight bias for SIDER2s in the divergent strand-switch regions of *L. major *and *L. braziliensis *can be observed (Tables 1 and 2). Scrutinizing the composition of strand-switch regions illustrates their conspicuous association to SIDER elements, as roughly 40% contain SIDERs on average (Table 2). Indeed, SIDER2s are 4- to 5-fold more abundant than SIDER1s in these locations and approximately 20-fold more for *L. major *in particular. There is also evidence that SIDER sequences may have been co-opted for protein-coding genes or pseudogenes in all three *Leishmania *species. In some cases, a start or stop codon intersects with a SIDER element. These hits to the refined HMM profiles tend to present lower than average bit-scores for the main part (data not shown). In agreement with our previous study [21], very little SIDERs locate to subtelomeric regions (as defined in the Methods section).

**Table 2:** Divergent strand-switch (dSS) and convergent strand-switch (cSS) regions enclosing SIDERs.

	***L. major***		***L. infantum***		***L. braziliensis***	
			
**Species**	dSS	cSS	dSS	cSS	dSS	cSS
SIDER1	1	1	5	4	7	5
SIDER2	25	18	25	23	32	21
Both	5	2	5	5	6	7

SS + SIDERs	31	21	35	32	45	33
Genomic SS regions	62	62	84	84	118	119

### Impact of widespread integration of SIDERs on genomic organization and plasticity

**Figure 4:**
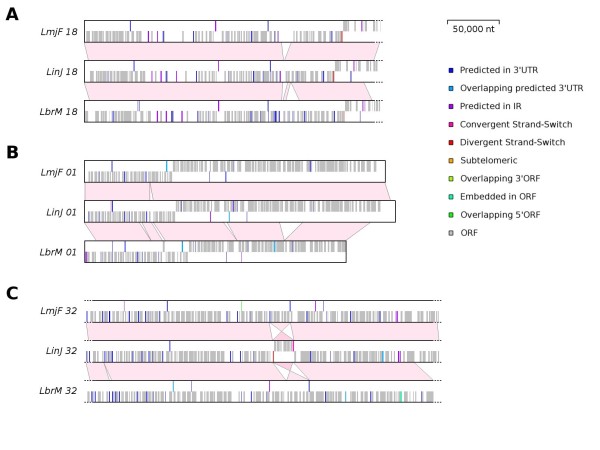
**Comparative chromosomal distribution of SIDERs in three *Leishmania *species**. Three examples of the comparative distribution of SIDER1 and SIDER2 sequences in *L. major *(LmjF), *L. infantum *(LinJ) and *L. braziliensis *(LbrM) are shown. Colored bars represent SIDERs, grey bars represent predicted open reading frames, and pink blocks illustrate syntenic relationships. **(A) **Segment of chromosome 18 where SIDER positions are synonymous in the three species. **(B) **Chromosome 1 displaying divergence in SIDER distribution amongst the three species. **(C) **Segment of chromosome 32 displaying both previous examples and an inversion of synteny flanked by SIDERs in *L. infantum*.

A large-scale comparative plot of the genomic positions of SIDER elements with regards to annotated coding sequences was performed for all three genomes (Additional file 5). The data confirm that SIDER elements of all types are quite uniformly dispersed throughout all three genomes. We identified three scenarios that highlight the impact of degenerated retroposons on the evolution of *Leishmania *genomes: (i) the genomic position of SIDERs is synonymous with that of the neighboring genomic locus in all three species (Figure 4A); (ii) SIDERs are located at different positions for at least one of the three species (Figure 4B); (iii) reorganization of DGC synteny (Figure 4C). The latter is an interesting example of chromosomal inversion with SIDER elements flanking the inverted region. This indicates that SIDERs continue to forge *Leishmania *genomes through homologous recombination events despite their extinct functions as transposable elements.

**Figure 5:**
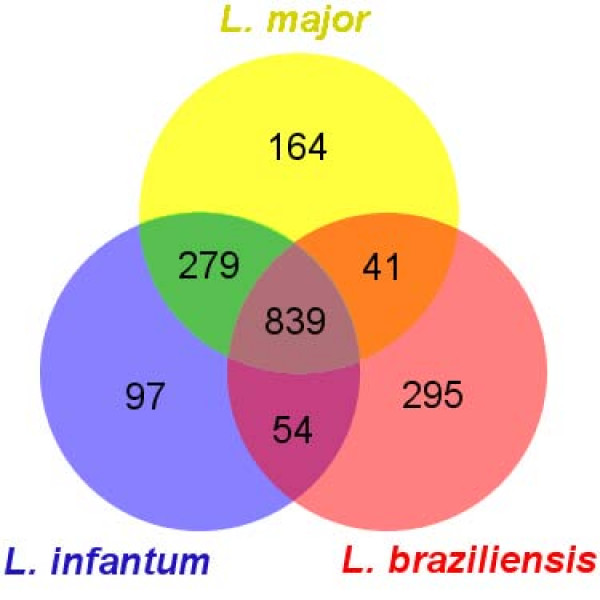
**Venn diagram of SIDER-associated *Leishmania *orthologs**. Numbers represent the exclusive amount of SIDER-associated orthologous genes shared between each species. Ortholog predictions were extracted from the *L. braziliensis *genome annotation (see text for details).

To further address the contribution of SIDERs to the genotypic diversity of *Leishmania *species, we investigated the association of these retroposons to orthologous genes in all three species. All *Leishmania *ortholog predictions included in version 2 (29/07/2008) of the *L. braziliensis *genome that present no more than one ortholog in *L. major *and *L. infantum *were extracted from GeneDB annotations [8]. The latter rely upon the *OrthoMCL *algorithm to predict all orthologs and recent segmental duplications (also referred to as in-paralogs) [37], thus ensuring that little or no genes are over-represented in the dataset. A total of 6802 orthologous genes were retained (*L. braziliensis *chromosome 20 was excluded from the analysis for technical reasons), of which 1769 (26%) are potentially associated to at least one SIDER element. Only 47.4% (839 out of 1769) of the orthologs harbor a SIDER element in all three species (Figure 5). When excluding these mutually shared retroposons, *L. major *and *L. infantum *share the second largest proportion of SIDER-associated orthologs (16%; 279 out of 1769), whereas *L. braziliensis *shares only 2.3% and 3% (41 and 54 out of 1769) of the SIDER-associated orthologs with either *L. major *or *L. infantum*, respectively. The observation that 31% of orthologs are associated to SIDERs in only one species and that another 21% harbor SIDERs in two out of three species supports the claim that these extinct retroposons may have undergone preferential assimilation or conservation in some species to some extent.

### Functional affiliations of SIDER elements

To determine if SIDER elements are preferentially associated to functionally related genes, we submitted the duplicate-free set of SIDER-associated orthologs to Gene Ontology (GO) term enrichment via the AmiGO browser available from the GeneDB website [8,38]. When excluding genes of unknown function, our findings indicate that both SIDER subfamilies are in some measure preferentially associated with genes belonging to similar functional categories (Additional file 6). When considering *P*-values inferior to 10^-2^, SIDER2 retroposons are preponderantly enriched in the 3'UTR of metabolic genes involved mainly in amino acid and amino acid derivative metabolic processes, nitrogen compound and amine biosyntheses cofactor and coenzyme metabolic processes, and carbon-carbon lyase and carboxylyase activities in the three *Leishmania *species tested. The SIDER1 subfamily is generally less associated to common functional groupings, yet *Li*SIDER1s present some evidence for enrichment in GO terms distinct from SIDER2s, namely cofactor binding and tetracycline transport (Additional file 6). Overall, the GO term enrichment analysis evokes that SIDER2-associated genes share some metabolic functions in all three species, which is not the case for SIDER1 elements that associate to genes with divergent GO terms.

The next question was to assess if and to what extent SIDER elements are ontologically enriched in a species-specific manner. The non-redundant *L. major *orthologs from all 7 categories depicted in Figure 5 were separated and submitted to GO term enrichment. Only SIDER-associated orthologs with assigned functions were retained for enrichment evaluation, the results of which are displayed in Table 3. The table summarizes the non-redundant GO terms in the sampled orthologs that present a *P*-value smaller than 0.01 when compared to the background set composed of all 4256 functionally annotated genes. It is apparent that orthologous mRNAs harboring SIDER elements in all three *Leishmania *species are associated with a variety of genes chiefly pertaining to metabolic processes and functions. There is also evidence for species-specific enrichment of GO terms in ortholog subsets where SIDERs are not present in all species. For instance, *L. major *and *L. infantum *but not *L. braziliensis *share SIDERs preferentially associated to genes involved in post-translational protein modification, phosphorylation, methylation, hydrolase activity acting on ester bonds, and carbon-sulfur lyase activity (Table 3). Conversely, some SIDERs are strictly associated to *L. braziliensis *orthologs with significantly related GO terms, notably cell cycle regulation, ribonucleoside metabolism, and aspartic-type endopeptidase activities. The remaining ortholog subsets comprise modest sample sizes, which may explain the lack of statistically significant enrichment.

**Table 3:** Gene ontology term enrichment of unique SIDER-associated orthologous *Leishmania *genes

Gene Ontology term	*P*-value	Fold enrichment^a^	Orthologs
**SIDER-associated genes in *L. major*, *L. infantum*, and *L. braziliensis *(439 sampled)**^b^			

*Biological Process*			
Nitrogen compound biosynthetic process	6.9E-6	3.8×	13
Cofactor metabolic process	4.1E-3	2.1×	14
Methionine metabolic process	9.3E-3	7.0×	3

*Molecular Function*			
Cofactor binding	3.7E-4	2.1×	23
Oxidoreductase activity	7.9E-4	1.6×	46
Acyl-CoA dehydrogenase activity	3.0E-3	4.5×	4
Vitamin binding	3.2E-3	2.3×	13

**SIDER-associated genes in *L. major *and *L. infantum *(163 sampled)**^b^			

*Biological Process*			
Post-translational protein modification	6.5E-3	1.8×	20

*Molecular Function*			
Hydrolase activity, acting on ester bonds	6.9E-3	2.0×	16
Phosphotransferase activity, alcohol group as acceptor	7.3E-3	1.8×	19
Carbon-sulfur lyase activity	8.3E-3	12×	2
Methyltransferase activity	9.3E-3	2.7×	8

**SIDER-associated genes in *L. major *and *L. braziliensis *(28 sampled)**^b^			

*n/a*			

**SIDER-associated genes in *L. infantum *and *L. braziliensis *(27 sampled)**^b^			

*n/a*			

**SIDER-associated genes in *L. major *only (94 sampled)**^b^			

*n/a*			

**SIDER-associated genes in *L. infantum *only (47 sampled)**^b^			

*n/a*			

**SIDER-associated genes in *L. braziliensis *only (156 sampled)**^b^			

*Biological Process*			
Ribonucleoside metabolic process	6.4E-3	6.3×	3
Regulation of mitotic cell cycle	7.6E-3	13×	2

*Molecular Function*			
Aspartic-type endopeptidase activity	6.4E-3	6.3×	3

^a ^[Sample frequency]/[Background frequency]

^b^Sampled genes exclude hypothetical proteins with no assigned function.

SIDER-associated orthologs were separated into 7 distinct subgroups for Gene Ontology term enrichment using AmigGO [38]. The unique corresponding *L. major *orthologs with annotated GO terms in each subgroup were sampled against a background of all functionally annotated genes in *L. major*. Only *P*-values ≤ 10^-2 ^(rmq: most of the values are between 10^-2 ^and 10^-3^) and over 1.5× enrichment are presented. *n/a*: not applicable.

## Discussion

The short interspersed degenerated retroposons of *Leishmania *present varying degrees of conservation and important fragmentation. This renders *in silico *analyses challenging, prospectively requiring exhaustive manual annotation of results. The use of HMM profiles renders the heterogeneous nature of a multiple sequence alignment into a comprehensive statistical model encoding both positional base composition and interactions between residues, hence reducing the subjectivity entailed by non-profile-based search strategies [39]. Version 1.8.5 of *HMMER *has the advantage of bearing powerful nucleotide alignment optimization algorithms on top of precise homology search tools. These reasons justify the use of *HMMER *over other programs such as *PSI-BLAST *or *meta-MEME *[40,41], although their effectiveness was not compared in this work. Nonetheless, HMMs have successfully been used to annotate TEs in other studies [42,43] and have also been shown to be more sensitive than pairwise or sequence-based methods [44-46]. The approach we advocate enabled the identification of sensibly the same amount of SIDER sequences in *L. major *as disclosed in our previous study [21], however distinct genome assembly versions may explain the subtle differences (version 4 vs. version 5.2). Slightly more than 100 SIDERs were identified in *L. infantum *and *L. braziliensis *genomes when compared to the 1858 previously annotated in *L. major*.

The relationship between chromosomal proximity and sequence similarity of SIDER2 repeats in *L. infantum *was recently put forward [34]. Requena *et al*. scrutinized the composition and arrangement of these elements on chromosomes 20 and 32, then compared the organization of SIDER2s on chromosome 32 of *L. braziliensis *and *L. major*. A laborious iterative *BLASTN *annotation strategy was conducted to uncover 27 *Li*SIDER2s on chromosome 20 and 54 on chromosomes 32. Using linear extrapolation, the authors estimated that around 1150 SIDER2s populate the *L. infantum *genome. In comparison, the automated profiling method employed in this work revealed 27 and 68 high scoring *Li*SIDER2s for the same chromosomes, respectively, and empirically annotated the position of 1236 *Li*SIDER2s. In the same article, the authors establish that *Li*SIDER2s bear only one of the two 79 nt signature sequences that characterize the 5' of *Lm*SIDER2s, and that the syntenic arrangement of SIDER2s is shared only between *L. major *and *L. infantum *(with regards to chromosome 32). The species-specific optimized HMM profiles we utilized clearly delineate the presence of two SIDER2 signature sequences in all three species (Figure 2). Furthermore, our results present the relatively conserved syntenic distribution of both SIDER subgroups for chromosome 32 and all the others (Figure 4 and Additional file 5). These observations further substantiate the use of profile-based approaches for SIDER alignment and genomic scanning. Nevertheless, our data for *L. major *concur with Requena *et al*. regarding the phylogenetic clustering of SIDER2s that correlates with chromosomal proximity (see phylogenetic tree in Additional file 1). This is not the case for the SIDER1 subgroup, besides the highly similar sequences related to gene duplications.

The remarkable expansion of SIDER sequences throughout *Leishmania *genomes in conjunction to their involvement in post-transcriptional regulatory processes [14,21] are evidence that these ancient retroposons underwent exaptation, or domestication, by their host genomes. This affirmation is consistent with the strikingly biased distribution of SIDERs within 3'UTRs (Table 1), as confirmed with recently published computational tools [35]. Our previous studies have demonstrated a role for SIDER2 elements in post-transcriptional control by promoting mRNA destabilization [21] and of SIDER1s in translational regulation [20,23]. The ability of transposable elements to contribute regulatory sequences to eukaryotic genomes is now becoming increasingly apparent [47-52]. SIDER2s in all three analyzed genomes appear to be preferentially associated to groups of genes implicated in common metabolic processes. It is therefore possible that these SIDER2-associated genes are regulated in a coordinated manner, for example in response to certain nutrient availabilities in their environment. *Leishmania *parasites are subjected to dynamic environmental changes within the phagolysosome, which have an immediate impact on their metabolic regulation [53]. Several examples of coordinated mRNA decay and multi-dimensional networks of post-transcriptional regulation have been reported in the budding yeast *S. cerevisiae *[54-56]. Experiments are now under way to test this hypothesis in *Leishmania*.

It has been shown that the 79 nt signature sequence from the distantly related L1Tc retroposon can promote transcription of the downstream sequence in *T. cruzi *[57]. Our data substantiate this finding since divergent strand-switch regions, known to be active transcriptional initiation points [10,11], frequently harbor SIDER elements (Tables 1 and 2). This outcome is also observed for other trypanosomatid retroposons [29]. Supporting the possibility that dSS SIDERs promote transcription initiation are several reports that attest to the profusion of TE-derived sequences in experimentally characterized human promoters [47,51,52]. Since 30 to 50% of strand-switch regions contain SIDER elements (Table 2), a considerable portion of these opposing DGCs may have originated from homologous recombination events prompted by SIDER-related repeats, including DIREs.

A manifest inversion of synteny that is flanked by SIDER elements in *L. infantum *(Figure 4C) demonstrates that short interspersed degenerated retroposons can be regarded as dynamic elements that play an ongoing role in genome plasticity and evolution. Also, since many SIDER-associated genes share comparable functions in all combinations of species investigated, such differential recruitment further exposes their contribution to genotypic diversity. On top of that, the observed enrichment of antisense SIDERs in interstitial pre-mRNA regions (between polyadenylation and *trans*-splicing sites) is quite intriguing (Table 1). One cannot rule out that highly conserved regions shared between sense and antisense SIDER RNA products could form RNA duplexes, which might play a role in SIDER-mediated regulation. It has been shown that TEs contribute tens of thousands of *cis *natural antisense transcripts to human genes [58-60]. The potential regulatory effects of these TE-derived antisense transcripts are substantial. For instance, we have recently shown that overexpression of antisense SIDER2 RNA can block SIDER2-mediated mRNA degradation (Müller *et al*., manuscript submitted for publication).

Our work clearly demonstrates that SIDER1 and SIDER2 belong to two distinct phylogenetic groups. It is also apparent that, when compared to SIDER2s, SIDER1 sequences are more heterogeneous and bear only the first 79 nt signature (Figures 1 and 2). If one considers that SIDER1 and SIDER2 elements most likely fulfill distinct regulatory functions [14,20,21,23] and that the second signature in SIDER2 is important for SIDER2-mediated mRNA degradation [Müller *et al*., manuscript submitted for publication], the lack of the second signature sequence in SIDER1 may be an evolutionary feat aiming at diversifying regulatory functions in these parasites. It is tempting to speculate that the degenerate nature of SIDERs can provide an abundant molecular staple from which *de novo cis*-regulatory elements emerge. Such a highly dynamic potential of regulation and adaptability is an effective strategy for a parasite to survive within its host.

Finally, our work demonstrates that the common ancestor of trypanosomatids contained one or more retroposons that are no longer active in present day *Leishmania *species [3]. Indeed, DIREs (and SIDERs) are the only vestiges of retroposons belonging to the *ingi *clade characterized in *Leishmania *genomes so far. In *L. major*, most of the *Lm*DIREs identified share their extremity or are in a close proximity to SIDER1 elements (Figure 3 and Additional file 3). Our experimental annotation of *Lm*SIDER1s did not exclude hits that overlapped with known DIRE elements, although the low frequency, large size, and poor conservation of DIRE retroposons in the genome would not significantly impact HMM modeling when contrasted to the over 15-fold higher abundance of SIDER1s. Consequently, we postulate that SIDER1 elements have derived from long active *ingi*-related retroposons in the ancestral *Leishmania *genome, as previously observed for the trypanosomatid RIME/*ingi *and NARTc/L1Tc non-autonomous/autonomous retroposon couples [61-63]. The main difference between these retroposon couples is that ingi and L1Tc are still active elements, whereas Leishmania genomes do not contain active retroposon family belonging to the ingi clade. Thus, the active retroposons that gave rise to SIDER1 is no longer active in the Leishmania genus as only vestiges have been identified (DIREs). No traces of a SIDER2 precursor have been detected so far, probably due to the evolutionary loss of corresponding DIRE sequences in the *L. major *genome. The possibility that such DIRE sequences have not yet been identified cannot be excluded, although the presence of DIREs in the genome of the two other *Leishmania *species has not been reported to date. The dissemination of SIDER predecessors in intergenic regions supplied *Leishmania *species with novel genetic material that contributed to increasing genomic plasticity and diversifying regulatory functions. Their assimilation most likely helped the parasite gain an auspicious evolutionary edge with regards to its complex parasitic lifestyle. Such a phenomenon is emerging as a widespread mechanism of assembly and tuning of gene regulatory systems in eukaryotes [64].

## Conclusion

The intra- and inter-genomic characterization of SIDER elements in three currently sequenced *Leishmania *genomes enabled the first direct characterization of SIDER1s, which are more heterogeneous than SIDER2s, lack the second 79 nt signature sequence of trypanosomatid retroposons and share sequence traits with DIRE elements. Our results confirm previous reports and establish that, in addition to the widespread localization of sense-oriented SIDERs in 3'UTRs, antisense SIDERs are enriched in interstitial regions between polyadenylation and *trans*-splicing sites. Our work also demonstrates that divergent strand-switch regions, proven to be involved in transcription initiation, frequently harbor SIDERs.

The comparative analysis of the distribution of SIDERs relative to protein-coding genes in three *Leishmania *species provides evidence that a common *Leishmania *ancestor was colonized by one or more active precursor retroposons. Albeit SIDERs are uniformly scattered throughout the genome, their integration is not random. SIDERs are to some extent preferentially associated with groups of genes encoding a similar biological function, which alludes to their potential role in coordinating gene regulation. The fact that SIDERs demonstrate species-specific associations to orthologous genes evokes their repercussion on genotypic diversity and possible contribution to species-specific gene expression. We propose that the abundance and diversity of SIDERs increased the plasticity of *Leishmania *genomes, providing the genus with molecular thread to weave fine-tuned regulatory fabric in response to the selective pressures arising from a complex digenetic parasitic life cycle.

## Methods

### SIDER retroposon alignment and phylogeny

Initial sequence data was obtained from version 5.2 of the *L. major *genome annotation, downloaded from the GeneDB FTP server [8]. All *Lm*SIDERs spanning between 350 and 700 nucleotides as annotated by Bringaud *et al*. [21] were extracted and used to perform multiple alignments using the *hmmt *program implemented in *HMMER-1.8.5 *software [65]. A first alignment optimization was produced using the default simulated annealing algorithm while specifying parameters *"-r 0.995 -k 10" *to override Viterbi refinement and the resulting alignment was submitted to guided Baum-Welch expectation maximization (parameters *"-B -i"*). Parsimonious informative columns of the final alignment were submitted to minimum evolution phylogeny as implemented in the *MEGA4 *program [66]. The phylogeny was modeled on p-distance while considering pairwise deletions. The multiple alignment representation was accomplished with *Jalview *[8].

### Generating selective profiles and iterative search strategy

*Lm*SIDER sequences were divided into two subgroups (SIDER1 and SIDER2) according to their phylogenetic relationship, then de-gapped and aligned independently using the same parameters as described above. Any sequence displaying over 90% sequence identity to another sequence was discarded, just the same as improperly labeled sequences (e.g., SIDER2 sequences in the SIDER1 cluster based on the previous manual annotation). The initial SIDER2 alignment was governed by a Hidden Markov Model (HMM) profile modeled from the published manual alignment in order to exploit its meticulous content, then resubmitted to Baum-Welch expectation maximization for sake of consistency. The initial HMM alignment profiles for *L. major *were used as a probe for genome-wide scans in all three sequenced *Leishmania *species. The *L. major *V5.2, *L. infantum *V3.0a, and *L. braziliensis *V2.0 genomes were downloaded from the GeneDB FTP server [8]. The Smith-Waterman-like fragment search (*hmmfs*) algorithm implemented in *HMMER-1.8.5 *was used to perform all queries. *Ad-hoc JAVA *scripts were used to concatenate fractionated hits which are characterized by insertions ≤ 150 nt and to classify hits as SIDER1 or SIDER2. Dichotomization of overlapping hits was based on the quotient of the SIDER1/SIDER2 bit-scores; a ratio <0.4 was identified as SIDER2, and a ratio >2.5 as SIDER1. Intervening ratios were assigned to the subfamily corresponding to the longest hit. The incorporation of search hits into species- and subclass-specific refined HMM profiles was governed by three essential conditions: (i) sequences must encompass 90% or more of the search profile's consensus; (ii) sequences must share less than 90% pairwise identity with any other sequence in the set of results; (iii) score over 50 bits. The resulting refined HMM profiles were aligned using the same parameters as described above before being used for final genomic scans.

### Genomic mapping of SIDERs

The prediction of polyadenylation and *trans*-splicing sites was performed using the *PRED-A-TERM *algorithm [35]. We define a strand-switch region as any intergenic sequence separated by two consecutive protein-coding genes that display different transcriptional orientations. We characterize subtelomeric regions as chromosomal extremities located >5 kb before or after terminal protein-coding sequences. SIDER positions were plotted on their respective chromosomes via an *ad-hoc *script and the *R *project for statistical computing [15]. All computational methodologies were carried out with an Intel q6600 processor overclocked to 3.4 GHz with 3GB RAM and Ubuntu Linux operating system.

## Authors' contributions

MS performed all analyses, helped design the study and contributed to the writing of the manuscript. FB validated results, assisted in the coordination of the study and helped compose the manuscript. BP conceived the study, coordinated many aspects of the study and contributed to the writing of the manuscript. All authors have read and approved the final manuscript.

## Supplementary Material

Additional file 1 **Alignment and phylogeny of 1416 *Lm*SIDERs**. Detailed representation of the data presented in Figure 1. The project can be opened and modified with the freely available *JALVIEW *software [8]. Click here for file

Additional file 2 **Selectivity scatter-plot of initial SIDER profiles**. Unaligned input sequences were scanned with the initial HMM profile of two SIDER subgroups using the *hmms *global alignment command from *HMMER-1.8.5*. The bit-scores for each sequence are plotted in the bidimensional grid. Click here for file

Additional file 3 **Linkage between *Lm*SIDER1 and *Lm*DIRE sequences**. All *Lm*DIREs identified in the *L. major *genome are represented by large black lines whereby the numbers correspond to the chromosome location and the order of appearance on the chromosome. The corresponding *Lm*SIDER1 sequences are shown above the DIRE line by a thin red line. Click here for file

Additional file 4 **All SIDER positions identified with refined HMM profiles in three *Leishmania *species**. Microsoft Excel spreadsheet containing all hits scoring over 0 bits with the refined HMM profiles reported in this work. Separate tabs for individual profiles. Click here for file

Additional file 5 **Comparative genomic distribution of SIDERs in three *Leishmania *species**. Complete distribution of SIDERs in all chromosomes of *L. major*, *L. infantum*, and *L. braziliensis*. Same description as in Figure 4. Click here for file

Additional file 6 **Gene Ontology term enrichment of SIDER1- and SIDER2-associated genes in all three *Leishmania *species**. Microsoft Excel spreadsheet displaying all results with *P*-values < 0.05 obtained with AmiGO term enrichment from GeneDB website (see text for details). Each tab encloses the data for one particular SIDER subgroup. Click here for file
