# Supplementary figures and images for: Organization and evolution of two SIDER retroposon subfamilies and their impact on the Leishmania genome
Source: BMC Genomics. 2009 May 22;10:240. doi: 10.1186/1471-2164-10-240 (PMC2689281; doi:10.1186/1471-2164-10-240)

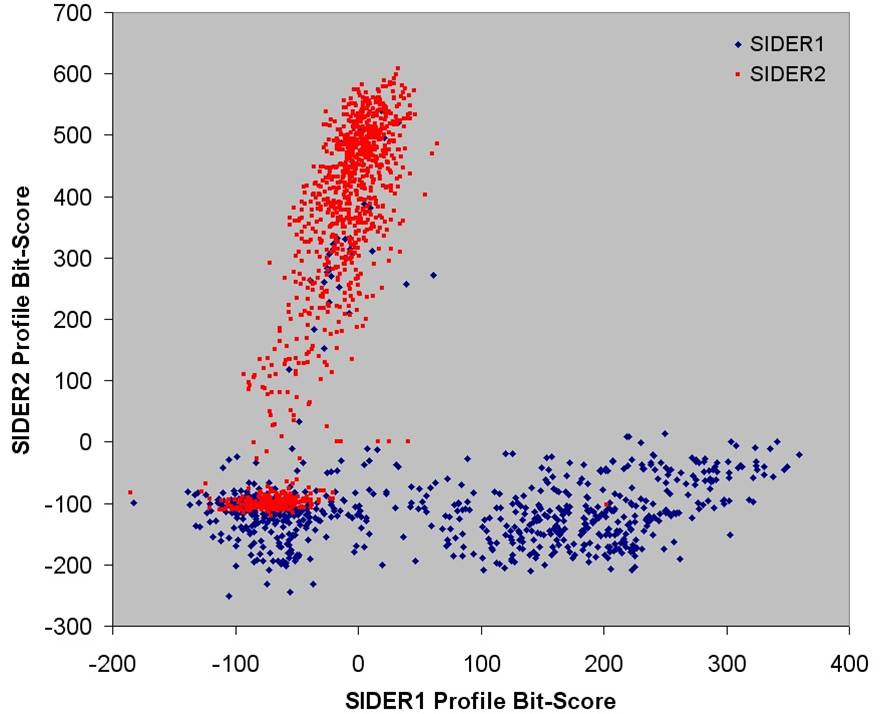

Supplement: Additional file 2 — Selectivity scatter-plot of initial SIDER profiles. Unaligned input sequences were scanned with the initial HMM profile of two SIDER subgroups using the hmms global alignment command from HMMER-1.8.5. The bit-scores for each sequence are plotted in the bidimensional grid. [file 1471-2164-10-240-S2.jpeg]

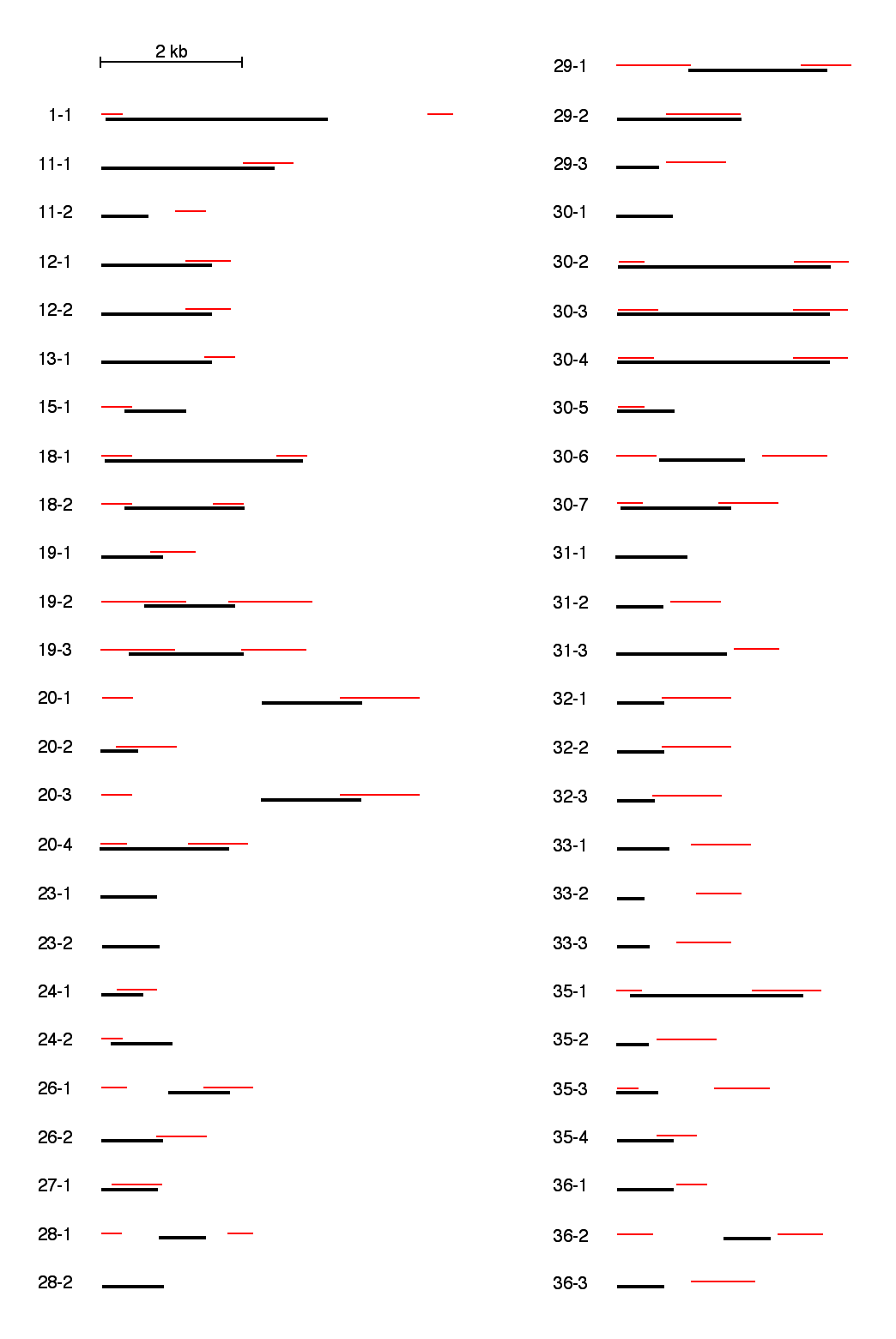

Supplement: Additional file 3 — Linkage between LmSIDER1 and LmDIRE sequences. All LmDIREs identified in the L. major genome are represented by large black lines whereby the numbers correspond to the chromosome location and the order of appearance on the chromosome. The corresponding LmSIDER1 sequences are shown above the DIRE line by a thin red line. [file 1471-2164-10-240-S3.png]

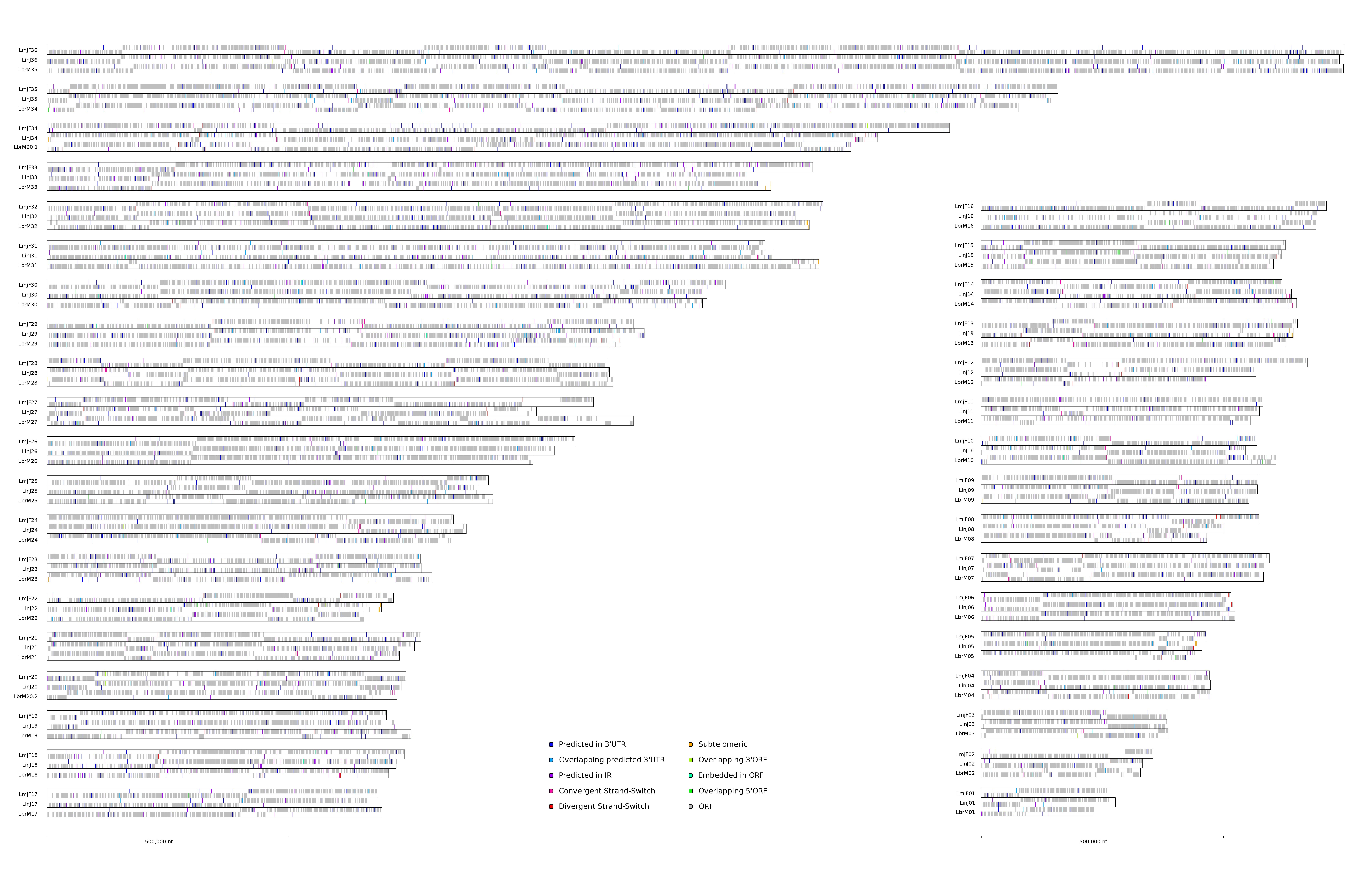

Supplement: Additional file 5 — Comparative genomic distribution of SIDERs in three Leishmania species. Complete distribution of SIDERs in all chromosomes of L. major, L. infantum, and L. braziliensis. Same description as in Figure 4. [file 1471-2164-10-240-S5.jpeg]
